# Methodological approaches for analysing data from therapeutic efficacy studies

**DOI:** 10.1186/s12936-021-03768-1

**Published:** 2021-05-21

**Authors:** Solange Whegang Youdom, Leonardo K. Basco

**Affiliations:** 1 Department of Public Health, Faculty of Medicine and Pharmaceutical Sciences, University of Dschang, P.O. Box 96, Dschang, Cameroon; 2 Aix-Marseille Universit, Institut de Recherche pour le Dveloppement (IRD), Assistance Publique Hpitaux de Marseille (AP-HM), Service de Sant des Armes (SSA), Unit Mixte de Recherche VecteursInfections Tropicales et Mditerranennes (VITROME), Marseille, France; 3 Institut Hospitalo-Universitaire (IHU)Mditerrane Infection, Marseille, France

**Keywords:** *Plasmodium falciparum*, Drug resistance, Artemisinin, Ordinal outcome, Multiple comparison, Network meta-analysis, Ranking, Analytics, Data science

## Abstract

Several anti-malarial drugs have been evaluated in randomized clinical trials to treat acute uncomplicated *Plasmodium falciparum* malaria. The outcome of anti-malarial drug efficacy studies is classified into one of four possible outcomes defined by the World Health Organization: adequate clinical and parasitological response, late parasitological failure, late clinical failure, early treatment failure. These four ordered categories are ordinal data, which are reduced to either a binary outcome (i.e., treatment success and treatment failure) to calculate the proportions of treatment failure or to time-to-event outcome for KaplanMeier survival analysis. The arbitrary transition from 4-level ordered categories to 2-level type categories results in a loss of statistical power. In the opinion of the authors, this outcome can be considered as ordinal at a fixed endpoint or at longitudinal endpoints. Alternative statistical methods can be applied to 4-level ordinal categories of therapeutic response to optimize data exploitation. Furthermore, network meta-analysis is useful not only for direct comparison of drugs which were evaluated together in a randomized design, but also for indirect comparison of different artemisinin-based combinations across different clinical studies using a common drug comparator, with the aim to determine the ranking order of drug efficacy. Previous works conducted in Cameroonian children served as data source to illustrate the feasibility of these novel statistical approaches. Data analysis based on ordinal end-point may be helpful to gain further insight into anti-malarial drug efficacy.

**Supplementary Information:**

The online version contains supplementary material available at 10.1186/s12936-021-03768-1.

## Background

Clinical efficacy of artemisinin-based combination therapy (ACT) has been monitored in many countries using the standard World Health Organization (WHO) protocol [1,4]. The following categorical outcome is used in WHO-recommended protocol: adequate clinical and parasitological response (ACPR), late parasitological failure, late clinical failure, and early treatment failure [1, 5]. The definitions of these four possible outcomes in *Plasmodium falciparum*-infected patients suggest that there is an explicit order in terms of disease severity and that these outcomes can be considered as ordinal variables. However, in most studies, this four-level primary outcome is reduced to a binary outcome (i.e., ACPR *versus* failure) to calculate the proportion of cured patients in per-protocol population [1, 6,8]. KaplanMeier curve analyses time-to-event outcomes to compensate partially for the loss of information, but data may be censored for various reasons (exclusion, withdrawal, loss-to-follow-up, clinical aggravation, or reinfection). These two methods of data interpretation may restrict the full exploitation of clinical results.

Several alternative approaches have been used to identify the most efficacious artemisinin-based combination that is useful to control and eliminate malaria [6]. This paper presents three approaches and areas of reflexion on how statistical analysis of existing database on anti-malarial drug efficacy can provide still unmined information. These approaches were presented in detail in previous works using data from therapeutic efficacy studies conducted in Cameroon [9,11]. They include (i) analysis of the ordinal outcome at a single time-point (day 14); (ii) analysis of the ordinal outcome over several time-points (day 14, day 21, and day 28); and (iii) network meta-analysis (NMA).

## Ordinal outcome with a single fixed time-point

Proportional odds models are regression models that may be more suitable to accommodate the 4-level ordinal outcome since these approaches take into consideration the order of the categories and adjust the models subsequently on either an agglomerated data or individual data. They can be used in different analytic settings and were found in some fields to provide greater power than time-to-event and binary endpoints [12]. The models are based on a logistic link function and account for both fixed and random effects, as well as baseline covariates [13]. However, difficulties may arise in the estimation process when some categories are not observed in a study. To circumvent this problem, the model has several extensions to account for categories with very few or no observations, especially when the efficacy of a test drug is very high (i.e.>90%). The complementary loglog link function provides suitable interpretation of results, which is similar to that of Cox-proportional hazard model.

Despite these limitations, proportional odds models have been recommended by some authors to combine the results of different types of clinical trials, including longitudinal and one-off studies, in which the outcome is categorically ordered [14, 15]. Ordinal logistic regression assumes that the coefficients that describe the relationship between each pair of outcomes are the same. This relationship is tested by a graphical method and likelihood comparison of the model with and without covariates [15, 16].

This approach was applied to clinical studies conducted in Cameroon at the time when the WHO was recommending a 14-day follow-up for areas of intense transmission [5, 9, 10]. Individual patient data were available. The interpretation of the proportional odds model accounts for the order across categories in the analytic setting. Categories can be ordered from the worst to the best or vice-versa. The odds ratio of comparison of drug A *versus* drug B is interpreted as either a progression towards success or progression towards failure [9]. Although the results of this retrospective study can no longer be of help for drug policy change since monotherapies are not used for the treatment of acute uncomplicated malaria, the application of the method illustrates the potential of alternative statistical methods. This method is illustrated in Additional File 1 using a simulated data set.

## Ordinal outcome with several time-points

The first approach was extended to data from *P. falciparum*-infected patients evaluated on days 14, 21, and 28 to account for time-effect in the ordinal regression model and correlation among individual responses to treatment [17]. In such cases, data analysis becomes more complex when multiple treatments are involved, leading to incomplete block design for treatment arms between different trials and the presence of variability. In addition, trials that are not directly connected to others are a potential source of wide variance and are removed from analysis [18, 19]. A statistical approach to handle an incomplete design is to use a proportional odds model where individual log-residual variance is modelled as a linear mixed model that accounts for time-covariate related to the outcome [20]. The effects of individual covariates at inclusion, such as parasitaemia, fever, sex, and weight, are important factors that may decrease heterogeneity across studies and should be accounted for.

The use of direct and indirect comparison has increased over recent years [21, 22]. Indirect comparison is performed using a common comparator drug between treatment regimens that are not linked because they were not compared in a randomized clinical study [10]. By contrast, direct comparison implies a direct link between two treatments with the number of studies comparing both treatments. Bonferroni correction is recommended and is used to estimate the common type 1 error for all comparisons, which results in large confidence intervals. Linear mixed models can be trickier in handling because of complex maximum likelihood that they generate and the difficulty in integrating the likelihood. Despite these difficulties in the formulation of prior distributions, Bayesian methods have been the most commonly used tests to compensate the look-elsewhere effect in such cases because of their flexibility.

To illustrate this approach, clinical studies conducted in Cameroon revealed that, compared to artesunate-amodiaquine (ASAQ), dihydroartemisinin-piperaquine (DHPP) was significantly more efficacious, i.e. there was a positive progression towards ACPR from day 14 to day 28 with DHPP, suggesting that more treatment failures occurred during day 14 and day 21 for ASAQ compared to DHPP [10]. Progression to success was similar between ASAQ and artemether-lumefantrine (AMLM), in agreement with other studies conducted in Africa [23,27]. The apparent superiority of DHPP was assessed in a larger patient population and is in agreement with pharmacokinetic profiles of amodiaquine and lumefantrine, which have shorter elimination half-lives than piperaquine [28].

## Network meta-analysis

NMA can be used to extend the second approach to multiple randomized clinical trials with the aim of choosing the best treatment regimen [29, 30]. The modelling process is based on random effect models that account for different sources of variability, including drug formulation, mode of administration, supervised *vs* unsupervised drug administration, conflicting results reported by each trial, and study design (multicentric *vs* single centre). When multiplicity is present, some authors opt for a frequentist approach, while others argue for the use of a Bayesian approach [31]. Both of these approaches can be implemented using available statistical packages [32], which are also suitable for single trial proportional model.

Data from randomized studies conducted in Cameroon, and elsewhere in Africa, have been combined to illustrate the utility of NMA using a binary outcome [11, 33]. These analyses showed that DHPP was more effective than AMLM (odds-ratio [OR]=1.92; 95% confidence interval [CI] 1.302.82; 19,163 patients) and that DHPP has the highest probability of being the best choice for treating uncomplicated *P. falciparum* malaria*.* A similar study was conducted in Asia, and the network built with 14 treatment regimens revealed that the OR network estimates from both African and Asian studies were comparable. In Asia, DHPP was 2.5 times (95% CI, 1.085.8) more efficacious on day 28 than AMLM [34].

One of the disadvantages of NMA is its high sensitivity to highly effective novel ACT in a small sample size because ranking may depend on whether the drug has been widely assessed or not [11]. This problem can be circumvented by ranking treatment regimens from the most tested ACT to the less tested ACT [11]. The methodological approach of ranking treatment also depends on whether a frequentist approach based on P-score and an analog to the surface under the cumulative ranking (SUCRA) or a Bayesian approach with posterior probability is used [35,37].

## Discussion

The ordinal criterion was applied to data from therapeutic efficacy studies for alternative statistical analysis and data interpretation [9,11]. Some authors have argued that ordinal regression models may be superior to analysis of binary outcome for designing clinical trial and evaluating treatment efficacy [12, 38]. In the present opinion paper, it is argued that ordinal data analysis may be helpful to evaluate fixed-time efficacy, or changes in therapeutic responses over time at the individual level where follow-up assessment allows detection of recrudescence/reinfection and evaluate the correlation between therapeutic response and molecular markers of resistance [39]. Modelling the WHO criteria as ordinal criteria results in a gain of information and precision on the estimate of the treatment effect. However, one of its limitations is the difficulty in determining the sample size of a trial given a known treatment effect [40].

KaplanMeier analysis is modelled based on the time to failure, reports the risk of recrudescence, and considers several events, such as reinfections, withdrawals, and loss-to-follow-up, as censored [8]. However, it does not consider categorical outcome. Since more robust statistical methods are available for handling categorical outcome [16], it is the opinion of the present authors that clinical studies on anti-malarial drug should account for such outcome since this innovative methodological approach provides information that has been missed heretofore and higher precision on the estimate of the treatment effect [12]. In the context of the emergence and spread of artemisinin resistance [41, 42], it may be expected that an increasing number of patients will respond with one of the failure categories, enabling the optimal use of this approach to compare the efficacy of ACT and identify the most effective drug.

For systematic reviews, NMA constitutes a powerful analytical approach to bring together multiple treatments which have not been compared directly in randomized controlled trials. Treatment ranking is solely possible using NMA, rendering this tool useful to identify the best treatment based on available evidence. NMA also provides a greater statistical precision through its incorporation of indirect evidence, which is not considered in pairwise meta-analysis.

## Conclusions

The four-level ordinal outcome derived from the WHO protocol can be better exploited using several statistical tools to analyse agglomerated or individual patient data from single trials or multi-centric trials in which two or more treatments are evaluated. These analytical tools include options for multiple comparison with fixed and mixed effects and determination of the best treatment regimen.

## Supplementary Information

**Additional file 1**: Illustration of data analysis with an ordinal outcome: WHO criteria for anti-malarial trial using a fixed effect model. Step by step analysis and interpretation of ordinal outcomes using R.

## Data Availability

Not applicable.

## Abbreviations

ACPR: Adequate clinical and parasitological response
ACT: Artemisinin-based combination therapies
AMLM: Artemether-lumefantrine
ASAQ: Artesunate-amodiaquine
95% CI: 95% Confidence interval
DHPP: Dihydroartemisinin-piperaquine
NMA: Network meta-analysis
OR: Odds-ratio
PCR: Polymerase chain reaction
SUCRA: Surface under the cumulative ranking
WHO: World Health Organization
