# Supplementary material for: Methodological approaches for analysing data from therapeutic efficacy studies
Source: Malar J. 2021 May 21;20:228. doi: 10.1186/s12936-021-03768-1 (PMC8139079; doi:10.1186/s12936-021-03768-1)
Supplement: Supplementary file 1 — Additional file 1: Illustration of data analysis with an ordinal outcome: WHO criteria for anti-malarial trial using a fixed effect model. Step by step analysis and interpretation of ordinal outcomes using R. [file 12936_2021_3768_MOESM1_ESM.docx]

Illustration of data analysis with an ordinal outcome: WHO criteria for antimalarial trial- Fixed effect model

Solange Whegang Youdom

3/12/2021

Step by step analysis and interpretation of ordinal outcomes

*# on R version 4.0.3 (2020-10-10)*

*# Data: malaria clinical data of three antimalarial drugs*

*# endpoint: day 14*

*# three centers*

*# Individual patient data available*

*# more reading: Whegang et al, BMC research methodology 2010*

*# Proportional odds models have been designed to agglomerate data*

*# or account for individual patient data*

Load the libraries

**require**(foreign)

**require**(ggplot2)

**require**(MASS)

**require**(Hmisc)

**require**(reshape2)

**library**(nlme)

**library**(MASS)

**library**(lattice)

**library**(base)

**library**(gee)

**library**(splines)

**library**(survival)

**library**(Matrix)

Load the data: the first part of the analysis uses an agglomerated data

Read in the data set

*#========= Using a grouped data ==========================================*

*# One can make use of the function polr in the MASS library*

*#=========================================================================*

*#==== PART I: DATA TRANSFORMATION*

*# set the treatment names*

x1=c("AQ" ,"SP", "AQ+SP")

*# Set a reference group AQ by creating dummy variables for the two others*

x=c(0,0,1) *# for AQSP compared to AQ*

y=c(0,1,0) *# for SP compared to AQ*

trt1=factor(rep(y,rep(4,length(y))) ) *# SP vs AQ*

trt2=factor(rep(x,rep(4,length(x))) ) *# ASSP vs AQ*

y=c("Yaoundé","Bertoua","Garoua")

Etude=factor(rep(y,rep(12,length(y))))

*# Create center/region/area covariate: create dummy variables*

centre1=ifelse(Etude=="Bertoua",1,0)

centre2=ifelse(Etude=="Garoua",1,0)

*# Create the WHO criteria*

z=c("ADCPR","LPF","LCF","ETF")

OMS=factor(rep(z,9))

freq=c(96.8,0,0,1.5,

86.8,3.2,0,9.8,

95.1,0,0,0,

89.6,3.4,0,0,

84.2,0,3.5,5.2,

90.1,1.6,0,0,

95,3.3,0,1.6,

89.6,0,0,5.1,

96.6,0,0,0)

*# Add the levels of transmission for each center/region/area*

*#level=factor(c(rep("High",24),rep("Low",12)) )*

Niv=factor(c(rep("High",24),rep("Low",12)) )

*# Recode the WHO criteria as an ordered factor*

*# Here we recoded from the best category A=ADCPR to the worst category D=ETF*

who_recode=as.factor( ifelse(OMS=="ADCPR","A",ifelse(OMS=="LTF","B",ifelse(OMS=="LCF","C","D"))) )

*# Combine the variables into one data frame*

tab2=data.frame(OMS, who_recode,trt1,trt2,Etude,centre1,centre2,Niv,freq)

*# Visualize the first 6 lines*

head(tab2)

## OMS who_recode trt1 trt2 Etude centre1 centre2 Niv freq

## 1 ADCPR A 0 0 Yaoundé 0 0 High 96.8

## 2 LPF D 0 0 Yaoundé 0 0 High 0.0

## 3 LCF C 0 0 Yaoundé 0 0 High 0.0

## 4 ETF D 0 0 Yaoundé 0 0 High 1.5

## 5 ADCPR A 1 0 Yaoundé 0 0 High 86.8

## 6 LPF D 1 0 Yaoundé 0 0 High 3.2

MODEL 1

*#========================== PART II ==============================*

*#→========== ADJUST THE MODEL FOR TREATMENT AND CENTER COVARIATES*

*#== method: logistic= calssical ordinal regression model*

WhiteH <- polr(who_recode ~ trt1+trt2+centre1+centre2, weights = freq, data = tab2, method="logistic")

## Warning in eval(family$initialize): non-integer #successes in a binomial glm!

s=summary(WhiteH)

##

## Re-fitting to get Hessian

s

## Call:

## polr(formula = who_recode ~ trt1 + trt2 + centre1 + centre2,

## data = tab2, weights = freq, method = "logistic")

##

## Coefficients:

## Value Std. Error t value

## trt11 1.07123 0.3833 2.79511

## trt21 -1.81845 0.8571 -2.12157

## centre1 -0.02057 0.3931 -0.05234

## centre2 -0.36978 0.4258 -0.86852

##

## Intercepts:

## Value Std. Error t value

## A|C 3.2339 0.3902 8.2877

## C|D 3.3375 0.3937 8.4768

##

## Residual Deviance: 306.2889

## AIC: 318.2889

Interpretation of coefficients

- An ordinal regression model with four categories will generate 3 intercepts. Treatment coefficients stand as logarithm of odds ratio.
- Parameters of interest:
  - trt11= 1.107 with t-value=2.88, suggests that patient progressed to the ACPR category with AQSP compared to AQ
  - trt21 -1.82027, with t-value=-2.11, suggests a progression towards negative categories with SP compared to AQ

MODEL 2

The complementary log-log link function is suitable when extreme category values occur. The log-log and complementary log-log links are the increasing functions F^-1(p) = -log(-log(p)) and F^-1(p) = log(-log(1-p)); some call the first the ‘negative log-log’ link. These correspond to a latent variable with the extreme-value distribution for the maximum and minimum, respectively. The WHO criteria can be considered as having an extreme value distribution, especially for PCR-adjusted outcome for failure. However, it can be challenging in fitting such a model on agglomerate data.

WhiteH <- polr(OMS ~ trt1+trt2+centre1+centre2, weights = freq, data = tab2,method="cloglog")

## Warning in eval(family$initialize): non-integer #successes in a binomial glm!

s=summary(WhiteH)

##

## Re-fitting to get Hessian

s

## Call:

## polr(formula = OMS ~ trt1 + trt2 + centre1 + centre2, data = tab2,

## weights = freq, method = "cloglog")

##

## Coefficients:

## Value Std. Error t value

## trt11 0.23416 0.1163 2.0140

## trt21 -0.43578 0.1674 -2.6036

## centre1 0.10071 0.1279 0.7873

## centre2 -0.05118 0.1331 -0.3845

##

## Intercepts:

## Value Std. Error t value

## ADCPR|ETF 1.1763 0.1207 9.7463

## ETF|LCF 1.4589 0.1276 11.4316

## LCF|LPF 1.5218 0.1295 11.7489

##

## Residual Deviance: 357.3422

## AIC: 371.3422

Interpretation with clog-log link

A proportional hazards model for grouped survival times can be obtained by using the complementary log-log link with grouping ordered by increasing time. Thus, interpretation of treatment coefficients in the second model is similar to the interpretation of hazard ratios.

The comparison of SP treatment to the reference treatment AQ, the parameter e-1.040 = 0.353, is interpreted as a hazards ratio which is assumed constant regardless of the response category. It suggests that the proportion of patients having positive response was lower with SP than with AQ. Between AQSP and AQ, the hazards ratio was e^1.80=6.05, suggesting an increase of positive responses with AQSP, compared to AQ.

Test the proportional odds assumption

mod.polr=WhiteH

x2<-logLik(mod.polr)

cat("Degrees of freedom Proportional Odds Logistic Regression \n")

print(df_of_polr_model <- attributes(x2)$df)

cat("Answering the question: Is proportional odds model assumption violated\n")

cat("P value for difference in AIC between POLR and Multinomial Logit model\n")

*# abs since the values could be negative. That is negative difference of degrees of freedom would produce p=NaN*

print(1-pchisq(abs(mod.polr$deviance-mod.multinom$deviance), abs(df_of_multinom_model-df_of_polr_model)))

Fitting the ordinal regression model with individual patient data

For individual patient data, several algorithms were developed to address this type of data. To the best of our knowledge, the software R does not yet offer this opportunity. These methods include the maximum likelihood approach, the iterative generalized least square, and the generalized estimation equation. In addition, Bayesian modelling of such data offers more flexibility, especially when one deals with a mixed effect model. We recommend interested readers to download materials from our additional files through the link <http://www.biomedcentral.com/1471-2288/10/58> : Whegang *et al*., Analysis of an ordinal outcome in a multicentric randomized controlled trial: application to a 3-arm anti-malarial drug trial in Cameroon. BMC Medical Research Methodology 2010, 10:58.

*#=========================================================================*

*#===== Load the various sub-programs written in a main file*

*# Make sure the directory is the appropriate one*

*#=========================================================================*

Check all the loaded libraries

**library**(nlme)

**library**(MASS)

**library**(lattice)

**library**(base)

**library**(gee)

**library**(splines)

**library**(survival)

**library**(Matrix)

Step 0: LOAD THE R CODES FOR THE THREE METHODS

They should be all stored in the same file (here a text file) and repository (here it is Document folder on local disk C), as the data to analyse. They will be called in Steps 6 and 7 to produce the parameter estimates.

*#=========================================================================*

*#===== Load various utilitary functions stored in UtilitairesR.txt.*

*# Make sure the directory is the appropriate one*

*#=========================================================================*

Location = "C:/Users/dess/Documents/"

Program = "MethodsRcodes.txt"

File = paste(Location,Program,sep="",collapse = NULL)

**source**(File)

*#=========================================================================*

*# Load the data from the appropriate directory.*

*# The data should have the extension .txt. The rows are constituted of*

*# subjects and the columns, of variables related to each subject.*

*#*

*# The data is the simulated example we studied in the current manuscript and*

*# is named Data.*

*# We simulated the data from the ordinal model containing only the treatment*

*# covariates. So the input values were p=(-2,-1,0,-1,1).*

*# We provided the csv file and the txt file.*

*#=========================================================================*

Step1: Read in the simulated dataset

This dataset’s column names have been formatted as required by the methods. Indeed, the aggregate data above have been used to simulate individual patient data.

directory = "C:/Users/dess/Documents/"

name_file = "simuldataR.txt"

fichier = sprintf("%s%s",directory,name_file)

data = read.table(fichier,header=TRUE,sep="\t",dec=".",na.strings="NA")

head(data)

## ID Etude Z Trt W1 W2 W3 W4 Y1 Y2 Y3 Y4 trt1 trt2

## 1 1 1 3 1 0 0 1 0 0 0 1 1 0 0

## 2 2 1 2 1 0 1 0 0 0 1 1 1 0 0

## 3 3 1 1 1 1 0 0 0 1 1 1 1 0 0

## 4 4 1 4 1 0 0 0 1 0 0 0 1 0 0

## 5 5 1 1 1 1 0 0 0 1 1 1 1 0 0

## 6 6 1 4 1 0 0 0 1 0 0 0 1 0 0

Step 2: Data filtering

Eliminate loss-to-follow-up and excluded patients from analysis; retain only those with a response category at the end-point. Doing this is similar to performing a per-protocol analysis. In an intention-to-treat approach, the user will need to input missing categories for these excluded children, at end-point.

nameVar=names(data)

nameVar

## [1] "ID" "Etude" "Z" "Trt" "W1" "W2" "W3" "W4" "Y1"

## [10] "Y2" "Y3" "Y4" "trt1" "trt2"

*#=========================================================================*

*# Eliminate lost to follow up and excluded subjects if any*

*# Corresponding codes (variable Z):*

*# PDV Lost to follow up subjects*

*# EXCLU Excluded subjects*

*#=========================================================================*

data=data[!data$Z=="EXCLU",]

data=data[!data$Z=="PDV",]

*#-------------------------------------------------------------------------*

*# data : new name of the working data.frame without the excluded and lost*

*# to follow-up subjects for subsequent analysis*

*#-------------------------------------------------------------------------*

Step 3: Create dummy variables and form the design matrix

*#=================================================================*

*# The objective is to test the treatment effects, taking into*

*# account the center effect*

*#=================================================================*

*#======================================*

*#=== The non-repeated design matrix*

*#======================================*

*#========================================================================================*

*# create the dummy variable from the 3 centers and 3 treatments. The center number one is*

*# considered as the reference center. Treatment 0 is considered as the reference.*

*#========================================================================================*

E2=ifelse(data$Etude==2,1,0)

E3=ifelse(data$Etude==3,1,0)

T1=data$trt1

T2=data$trt2

n=nrow(data)

vec=rep(1,n)

X2=data.frame(vec,T1,T2,E2,E3) *# the design matrix*

head(X2)

## vec T1 T2 E2 E3

## 1 1 0 0 0 0

## 2 1 0 0 0 0

## 3 1 0 0 0 0

## 4 1 0 0 0 0

## 5 1 0 0 0 0

## 6 1 0 0 0 0

Step 4: Replicate the data

Here, a row of an individual is repeated as the number of response categories minus one.

*#=========================================================================*

*# Here, replicate the data according to the number of intercept*

*# for an easy use in the GEE and IGLS approaches*

*#=========================================================================*

m=3

r=n*m

L=n

*#======================================*

*# Now repeat the design matrix X2*

*#======================================*

data1=data.frame(data,E2,E3)

*#------------------------------------------------------*

*# data 1 can be kept as a final replicated data set*

*# using the following command*

*#------------------------------------------------------*

*# Output = paste(Location,"DataModif.txt",sep="",collapse=NULL)*

*# write.table(data1,Output,quote=FALSE,sep=" ",na="NA",dec=".",eof="\n")*

head(data1)

## ID Etude Z Trt W1 W2 W3 W4 Y1 Y2 Y3 Y4 trt1 trt2 E2 E3

## 1 1 1 3 1 0 0 1 0 0 0 1 1 0 0 0 0

## 2 2 1 2 1 0 1 0 0 0 1 1 1 0 0 0 0

## 3 3 1 1 1 1 0 0 0 1 1 1 1 0 0 0 0

## 4 4 1 4 1 0 0 0 1 0 0 0 1 0 0 0 0

## 5 5 1 1 1 1 0 0 0 1 1 1 1 0 0 0 0

## 6 6 1 4 1 0 0 0 1 0 0 0 1 0 0 0 0

Step 5: Create a dataframe with covariate and response categories

The idea of the step is to format a design matrix to enable the run of algorithms.

T1rep=rep(data1$trt1,rep(m,length(data1$trt1)))

T2rep=rep(data1$trt2,rep(m,length(data1$trt2)))

U2=rep(data1$E2,rep(m,length(data1$E2)))

U3=rep(data1$E3,rep(m,length(data1$E3)))

U=data.frame(T1rep,T2rep,U2,U3)

ter=data1[,c(9,10,11)] *# extract the set of binary responses*

t=t(ter)

Res=c(t[1:nrow(t),])

dat=data.frame(U,Res) *# Data frame without the covariates representing the*

*# intercepts*

vec1=diag(3)

terme_nul=matrix(rep(vec1,n),ncol=3,byrow=T)

colnames(terme_nul)=c("alpha1","alpha2","alpha3")

*#==================================================*

*# The final repeated design matrix*

*#==================================================*

X=data.frame(terme_nul,U)

head(X)

## alpha1 alpha2 alpha3 T1rep T2rep U2 U3

## 1 1 0 0 0 0 0 0

## 2 0 1 0 0 0 0 0

## 3 0 0 1 0 0 0 0

## 4 1 0 0 0 0 0 0

## 5 0 1 0 0 0 0 0

## 6 0 0 1 0 0 0 0

*# alpha1, alpha2, and alpha3, are the 3 intercepts generated by the m=4 WHO categories*

Step 6: ESTIMATION METHODS

*#=====================================================================================#*

*# Frequentist approaches: Fixed Effect Models*

*# IGLS: First program*

*# IGLS: Second program*

*# ML : Third program*

*#=====================================================================================#*

These programs can be obtained under request to the authors of the article.

METHOD 1: IGLS: iterative generalized least squared

*# First program to run: IGLS*

*#*

*#-------------------------- The IGLS approach; this takes few minutes------------------*

*# This function returns the estimates, the number of iteration at convergence,*

*# the covariance-matrix, and the standard error*

iter4=iterative_prg_tous_IGLS(data=data1,U=U,dat=dat,X2=X2,X=X,param_0=c(-1,0,1,0,0,0.3,0.2),

n=n,m=m,r=r,L=L,epsilon=.Machine$double.eps^0.25,n_iter=500)

est=iter4[[1]][[1]] *# extract the estimates*

niter=iter4[[1]][[2]] *# the number of iteration at convergence*

matcovIGLS=iter4[[2]] *# extract the covariance matrix of the estimates*

sd=iter4[[3]] *# extract their corresponding standard errors*

METHOD 2: Generalized estimating equation

*# Second program to run : GEE*

*#-------------------------- The GEE approach; this takes few minutes--------------------*

*# The function returns only the estimates and the number of iteration at convergence*

*#---------------------------------------------------------------------------------------*

iter2=iterative_prg2(data=data1,U=U,dat=dat,X2=X2,X=X,param_0=c(-1,0,1,0,0,0,0),

n=n,m=m,r=r,L=L,epsilon=.Machine$double.eps^0.25,n_iter=500)

beta_GEE=iter2[[1]] *# extract the estimates*

*#=============================================================================*

*# At convergence, we obtain beta_GEE for which we need*

*# a covariance matrix, which is obtained by running the function cov_param*

*#=============================================================================*

*# run cov-param after extracting the estimates from iterative_prg2*

mat=cov_param(data=data,U=U,dat=dat,X2=X2,X=X,beta_GEE=beta_GEE,n=n,m=m,r=r,L=L)

mat_cov_GEE=mat[[2]]

SQRT_beta_GEE=mat[[3]] *# the corresponding standard error for the GEE estimates*

niter2=iter2[[2]]

METHOD 3: APPROACH 1- Maximum likelihood with Logit Link function

*# Third program to run : ML*

*#=============================================================================*

*# Maximum likelihood approach*

*# Here several optimization methods are proposed : BFGS, Nelder-Mead, CG...*

*#=============================================================================*

*#================================================================================*

*# For the maximum likelihood technique, we do not need a replication of the data*

*# We construct a data.frame of the potential covariates*

*#================================================================================*

*#------------------------ ML approach with the Logit link function---------*

*# Recall X2*

X2=data.frame(vec,T1,T2,E2,E3)

Poids_W=data.frame(data$W1, data$W2, data$W3, data$W4)

iter3=optim(p=c(-1,0,1,0,0,0,0),ML,method="BFGS",control=list(trace=1,maxit=20000),X2=X2,

Poids_W=Poids_W,m=3,n=n,hessian=TRUE)

## initial value 616.018443

## iter 10 value 516.413649

## final value 516.320054

## converged

*#===============================================================================#*

*# Extract the estimates and their standard errors #*

*#===============================================================================#*

Par_EMV=iter3$par

MLvalue=iter3$value *# This returns the maximum likelihood value*

Hessian=iter3$hessian

covparam=solve(Hessian)

ect_par=sqrt(diag(covparam))

METHOD 3: APPROACH 2- Maximum likelihood with clog-log link function

*#------------------ML approach with the cloglog link function---------------*

iter31=optim(p=c(-1,0,1,0,0,0,0),MLcloglog,method="BFGS",control=list(trace=1,maxit=20000),

X2=X2,Poids_W=Poids_W,m=3,n=n,hessian=TRUE)

## initial value 871.547037

## iter 10 value 516.873492

## final value 516.678107

## converged

*#===============================================================================#*

*# Extract the estimates and their standard errors #*

*#===============================================================================#*

Par_EMV1=iter31$par

MLvalue1=iter31$value

Hessian1=iter31$hessian

mlvalue1=iter31$value

covparam1=solve(Hessian1)

ect_par1=sqrt(diag(covparam1))

matr=matrix(0,8,7)

matr[1,]=iter4[[1]][[1]]

matr[2,]=iter4[[3]]

matr[3,]=iter2[[1]]

matr[4,]=mat[[3]]

matr[5,]=iter3$par

matr[6,]=ect_par

matr[7,]=iter31$par

matr[8,]=ect_par1

matresult=as.data.frame(t(matr))

rownames(matresult)=c("alpha1","alpha2","alpha3","Trt1-Trt0","Trt2-Trt0","Center2-Center1","Center3-Center1")

colnames(matresult)=c("IGLS"," ","GEE"," ","ML logit"," ","ML cloglog"," ")

Step 7: FINAL ESTIMATES FROM THE METHODS

*#===============================================================================*

*# Now one can run the different steps and find the results in the work directory*

*#===============================================================================*

THESE ARE THE FINAL RESULTS THAT WILL BE FOUND IN THE WORKING DIRECTORY INITIALLY CREATED BY THE USER.

fit=list()

fit$title <- "FREQUENTIST APPROACHES OF A FIXED ORDINAL MODEL"

fit$Model="Treatment and center covariates"

fit$Methods= "IGLS, GEE, ML logit, ML cloglog"

fit$Comments="IGLS and GEE are contructed on the logit link function"

fit$IGLS="iterative generalized least squares"

fit$GEE="Generalized estimating equations"

fit$MLlogit= "Maximum likelihood using the logit link function"

fit$MLcloglog= "Maximum likelihood using the cloglog link function"

fit$CovarianceMatrixIGLS=iter4[[2]]

fit$CovarianceMatrixGEE=mat[[2]]

fit$CovarianceMatrixMLlogit=covparam

fit$CovarianceMatrixMLcloglog=covparam1

fit$NumberOfIterationIGLS=iter4[[1]][[2]]

fit$NumberOfIterationGEE=iter2[[2]]

fit$LikelihoodMLlogit<-iter31$value

fit$LikelihoodMLcloglog<-iter3$value

fit$Results<- "Estimates for each method and their corresponding standard deviations"

*# ESTIMATES FROM EACH METHOD and Standard errors*

fit$Matresults<-matresult

print(fit)

## $title

## [1] "FREQUENTIST APPROACHES OF A FIXED ORDINAL MODEL"

##

## $Model

## [1] "Treatment and center covariates"

##

## $Methods

## [1] "IGLS, GEE, ML logit, ML cloglog"

##

## $Comments

## [1] "IGLS and GEE are contructed on the logit link function"

##

## $IGLS

## [1] "iterative generalized least squares"

##

## $GEE

## [1] "Generalized estimating equations"

##

## $MLlogit

## [1] "Maximum likelihood using the logit link function"

##

## $MLcloglog

## [1] "Maximum likelihood using the cloglog link function"

##

## $CovarianceMatrixIGLS

## [,1] [,2] [,3] [,4] [,5]

## [1,] 0.05213055 0.04014557 0.03563991 -0.0218457308 -0.0270439458

## [2,] 0.04014557 0.04265740 0.03712537 -0.0226985120 -0.0242126133

## [3,] 0.03563991 0.03712537 0.04095406 -0.0248149894 -0.0213994984

## [4,] -0.02184573 -0.02269851 -0.02481499 0.0575160031 0.0223809812

## [5,] -0.02704395 -0.02421261 -0.02139950 0.0223809812 0.0461901838

## [6,] -0.02355920 -0.02403486 -0.02448903 0.0007107525 -0.0005492134

## [7,] -0.02297071 -0.02385185 -0.02473739 0.0013605402 -0.0011093025

## [,6] [,7]

## [1,] -0.0235591997 -0.022970713

## [2,] -0.0240348635 -0.023851851

## [3,] -0.0244890297 -0.024737387

## [4,] 0.0007107525 0.001360540

## [5,] -0.0005492134 -0.001109303

## [6,] 0.0493381470 0.024301494

## [7,] 0.0243014938 0.050538717

##

## $CovarianceMatrixGEE

## [,1] [,2] [,3] [,4] [,5]

## [1,] 0.05327632 0.04121498 0.03666121 -0.022864254 -0.0275184610

## [2,] 0.04121498 0.04380010 0.03819827 -0.023600647 -0.0247214759

## [3,] 0.03666121 0.03819827 0.04210148 -0.026153888 -0.0218697028

## [4,] -0.02286425 -0.02360065 -0.02615389 0.062395680 0.0234569133

## [5,] -0.02751846 -0.02472148 -0.02186970 0.023456913 0.0467853594

## [6,] -0.02445036 -0.02491166 -0.02536997 0.000681110 -0.0006146882

## [7,] -0.02374142 -0.02466504 -0.02565008 0.001389057 -0.0013390875

## [,6] [,7]

## [1,] -0.0244503640 -0.023741423

## [2,] -0.0249116617 -0.024665045

## [3,] -0.0253699656 -0.025650077

## [4,] 0.0006811100 0.001389057

## [5,] -0.0006146882 -0.001339087

## [6,] 0.0512312976 0.025190259

## [7,] 0.0251902590 0.052824042

##

## $CovarianceMatrixMLlogit

## [,1] [,2] [,3] [,4] [,5]

## [1,] 0.05314484 0.04115953 0.03649825 -0.022168792 -0.0267493825

## [2,] 0.04115953 0.04360545 0.03792134 -0.022970015 -0.0240689791

## [3,] 0.03649825 0.03792134 0.04152447 -0.024971646 -0.0212663551

## [4,] -0.02216879 -0.02297001 -0.02497165 0.057873770 0.0224565098

## [5,] -0.02674938 -0.02406898 -0.02126636 0.022456510 0.0461157955

## [6,] -0.02459364 -0.02511369 -0.02532171 0.001474147 -0.0007866325

## [7,] -0.02389933 -0.02455167 -0.02519649 0.001091329 -0.0014591909

## [,6] [,7]

## [1,] -0.0245936400 -0.023899329

## [2,] -0.0251136909 -0.024551669

## [3,] -0.0253217121 -0.025196486

## [4,] 0.0014741475 0.001091329

## [5,] -0.0007866325 -0.001459191

## [6,] 0.0493086847 0.025134525

## [7,] 0.0251345251 0.051569553

##

## $CovarianceMatrixMLcloglog

## [,1] [,2] [,3] [,4] [,5]

## [1,] 0.03448276 0.02443965 0.02071148 -0.0128896890 -0.0147638793

## [2,] 0.02443965 0.02531301 0.02104703 -0.0130886440 -0.0142406873

## [3,] 0.02071148 0.02104703 0.02191021 -0.0135892915 -0.0134484035

## [4,] -0.01288969 -0.01308864 -0.01358929 0.0390000618 0.0131606833

## [5,] -0.01476388 -0.01424069 -0.01344840 0.0131606833 0.0225598610

## [6,] -0.01311182 -0.01310631 -0.01289924 0.0001067747 0.0007899185

## [7,] -0.01257828 -0.01264538 -0.01282015 0.0006733972 0.0002481492

## [,6] [,7]

## [1,] -0.0131118190 -0.0125782767

## [2,] -0.0131063130 -0.0126453841

## [3,] -0.0128992423 -0.0128201468

## [4,] 0.0001067747 0.0006733972

## [5,] 0.0007899185 0.0002481492

## [6,] 0.0256066091 0.0125289554

## [7,] 0.0125289554 0.0273773272

##

## $NumberOfIterationIGLS

## [1] 7

##

## $NumberOfIterationGEE

## [1] 6

##

## $LikelihoodMLlogit

## [1] 516.6781

##

## $LikelihoodMLcloglog

## [1] 516.3201

##

## $Results

## [1] "Estimates for each method and their corresponding standard deviations"

##

## $Matresults

## IGLS GEE ML logit

## alpha1 -1.8660271 0.2283212 -1.8696646 0.2308166 -1.8660277 0.2305316

## alpha2 -0.7660570 0.2065367 -0.7617439 0.2092847 -0.7660571 0.2088192

## alpha3 0.2274705 0.2023711 0.2374348 0.2051865 0.2274727 0.2037756

## Trt1-Trt0 -1.0640760 0.2398249 -1.0583584 0.2497913 -1.0640821 0.2405697

## Trt2-Trt0 1.0117774 0.2149190 1.0056781 0.2162992 1.0117770 0.2147459

## Center2-Center1 -0.2030695 0.2221219 -0.1926889 0.2263433 -0.2030703 0.2220556

## Center3-Center1 -0.3941727 0.2248082 -0.4085288 0.2298348 -0.3941745 0.2270893

## ML cloglog

## alpha1 -1.90949425 0.1856953

## alpha2 -0.98204346 0.1591006

## alpha3 -0.24885440 0.1480210

## Trt1-Trt0 -0.85747873 0.1974843

## Trt2-Trt0 0.70000587 0.1501994

## Center2-Center1 -0.04748565 0.1600207

## Center3-Center1 -0.27794009 0.1654610

Print the final result matrix

**library**(knitr)

**library**(kableExtra)

results=as.data.frame(fit$Matresults)

results=round(results, 3)

kable(results) %>% kable_styling(bootstrap_options=c("striped"),font_size=10,position="center")

| Parameters | **IGLS** | SE | **GEE** | SE | **ML logit** | SE | **ML cloglog** | SE |
| --- | --- | --- | --- | --- | --- | --- | --- | --- |
| alpha1 | -1.866 | 0.228 | -1.87 | 0.231 | -1.866 | 0.231 | -1.909 | 0.186 |
| alpha2 | -0.766 | 0.207 | -0.762 | 0.209 | -0.766 | 0.209 | -0.982 | 0.159 |
| alpha3 | 0.227 | 0.202 | 0.237 | 0.205 | 0.227 | 0.204 | -0.249 | 0.148 |
| Trt1-Trt0 | -1.064 | 0.24 | -1.058 | 0.25 | -1.064 | 0.241 | -0.857 | 0.197 |
| Trt2-Trt0 | 1.012 | 0.215 | 1.006 | 0.216 | 1.012 | 0.215 | 0.7 | 0.15 |
| Center2-Center1 | -0.203 | 0.222 | -0.193 | 0.226 | -0.203 | 0.222 | -0.047 | 0.16 |
| Center3-Center1 | -0.394 | 0.225 | -0.409 | 0.23 | -0.394 | 0.227 | -0.278 | 0.165 |

References

Whegang Youdom S, Basco LK, Gwét H, Thalabard JC. Analysis of an ordinal outcome in a multicentric randomized controlled trial: application to a 3-arm anti-malarial drug trial in Cameroon. BMC Med Res Method 2010;10:58.

Whegang Youdom S, Samson A., Basco LK, Thalabard JC. Multiple treatment comparisons in a series of antimalarial trials with an ordinal primary outcome and repeated treatment evaluations. Malar J. 2012;11:147.
